# Pharmacokinetics and Bioequivalence of Rasagiline Tablets in Chinese Healthy Subjects Under Fasting and Fed Conditions: An Open, Randomized, Single-Dose, Double-Cycle, Two-Sequence, Crossover Trial

**DOI:** 10.3389/fphar.2020.571747

**Published:** 2020-12-03

**Authors:** Yinjuan Li, Lu Qi, Haihong Bai, Ying Liu, Rongxia Fan, Yongrui Tu, Yongqiang Sun, Juxiang Wang, Qi Qi, Xiaohui Feng, Da Zhou, Xinghe Wang

**Affiliations:** ^1^ Department of Phase I Clinical Trail Center, Beijing Shijitan Hospital, Capital Medical University, Beijing, China; ^2^ Changzhou Siyao Pharmaceuticals Co., Ltd., Jiangsu, China

**Keywords:** bioequivalence, rasagiline, pharmacokinetic, food effect, highly variable drug

## Abstract

**Objective:** This study evaluated the pharmacokinetics, safety, and bioequivalence (BE) of two formulations of rasagiline tablets in healthy Chinese subjects under fasting and fed conditions.

**Methods:** An open, randomized, single-dose, double-cycle, two-sequence, self-crossover pharmacokinetic study in healthy Chinese subjects under fasting and high-fat postprandial conditions was performed. A total of 108 healthy subjects (36 in the fasting group and 72 in the postprandial group) were recruited. In each cycle of the study under both conditions, subjects received a single oral dose of 1 mg of a test or reference preparation of rasagiline tablets (1 mg each). A washout period of 3 days was observed. Blood samples were obtained up to 10 h post-intake. Primary endpoints were the BE of major pharmacokinetic parameters (AUC_0–t_ and AUC_0–∞_) and the maximum observed serum concentration (C_max_). Secondary endpoints were safety parameters.

**Results:** The 90% confidence interval (CI) of the geometric mean ratio (GMR) of the test drug vs. the reference drug for rasagiline was 94.16–105.35% for the AUC_0–t_ under fasting conditions and 99.88–107.07% under postprandial conditions. The 90% CIs for the AUC_0–∞_ were 93.55–105.01% and 99.59–107.05% under fasting and postprandial conditions, respectively. The 90% CIs for the C_max_ were 88.26–108.46% and 89.54–118.23% under fasting and postprandial conditions, respectively. The 90% CIs for the test/reference AUC ratio and C_max_ ratio were within the acceptable range (0.80–1.25) for BE. In this BE study, there were no serious adverse events (AEs).

**Conclusion:** BE between the test and the reference products was established in both fasting and postprandial conditions. The two formulations of rasagiline showed good tolerability and a similar safety profile.

**Clinical Trial Registration:** chinaDrugtrials.org.cn, identifier CTR20181466.

## Introduction

Parkinson’s disease (PD) is a progressively disabling neurodegenerative disorder caused by the loss of dopaminergic neurons in the substantia nigra pars compacta. Second only to Alzheimer’s disease (AD), PD is among the most common neurodegenerative diseases in the world, affecting approximately 0.3% of the general population and 1–3% of people above 65 years of age, and the number of people suffered from PD is predicted to climb increase from 8.7 to 9.3 million by 2030 (Raza et al., 2019). Currently, PD affects millions of people globally and has severe social and economic impacts.

The inhibitors of monoamine oxidase type B (MAO-B), which is the major enzyme responsible for the oxidative metabolism of dopamine in the human brain, can control the symptoms of PD. The first (MAO-B) inhibitor was selegiline, with a major drawback of metabolism to (−)-amphetamine and (−)-methamphetamine. These metabolites play an “anti-tyramine” role by inhibiting the uptake of dopamine by neurons, resulting in neurotoxicity and cardiovascular adverse effects (Avila et al.,2019; Chen and Swope, 2005; Finberg, 2020; Thébault et al., 2004). Rasagiline mesylate is a potent, highly selective, and irreversible second-generation MAO-B inhibitor and has neuroprotective activity (Chen and Swope, 2005; Teva Pharmaceuticals USA, 2018). Its major metabolite is 1(R)-aminoindan, which is differentiated by its distinctly non-amphetamine structural features (Thébault et al., 2004). Rasagiline mesylate (Azilect^®^), developed jointly by Israel’s Teva and Denmark’s Lundbeck, was first approved by the European Medicines Agency (EMA) in February 2005 and then approved by the US Food and Drug Administration (FDA) in 2006 as a monotherapy in PD patients not treated with levodopa and as an adjunct therapy to levodopa in levodopa-treated patients. In June 2017, rasagiline was approved by the CFDA to be listed in China and is currently used in over 50 countries by patients taking doses ranging between 0.5 mg and 1 mg daily (Finberg, 2020; Elgart et al., 2019).

According to recent surveys conducted with healthy individuals and PD patients in China and Japan, rasagiline can safely and effectively treat PD symptoms by blocking the decomposition of the neurotransmitter dopamine, with good drug tolerance and long-lasting effects (Chen et al., 2016; Elgart et al., 2019; Hattori et al., 2018; Zhang et al.,2018). In general, rasagiline is a novel, safe and effective drug for the management of PD.

Other than limited generic drugs, only two kinds of MAO-B inhibitors (rasagiline and safinamide) have been successfully commercialized. Consequently, PD patients have limited choices in these drugs (Avila et al., 2019). Although antiparkinsonian medications are not considered to be the most expensive pharmacological agents, lifelong treatment and often complex drug regimens impose a high economic burden on both patients and the healthcare system (Pintér et al., 2019). Accordingly, the development and application of generic drugs as substitutes for brand-name drugs is a clear and economical choice.

Rasagiline mesylate, produced by Changzhou Siyao Pharmaceuticals Co., Ltd. in tablet form, is the first generic drug of its type in China. Taking this test preparation and using Azilect^®^ as a reference, we applied a 2-sequence and 2-period crossover (2 × 2) study design to investigate the pharmacokinetic (PK) properties of rasagiline mesylate with single-dose administration in healthy Chinese volunteers under fasting or postprandial conditions and to assess the bioequivalence (BE) of the 2 rasagiline mesylate tablets after the administration of a single 1 mg oral dose to this population. This study was registered and approved by the China Food and Drug Administration.

## Materials and Methods

This study was conducted in full accordance with the Declaration of Helsinki and Good Clinical Practice guidelines and applicable national and local laws and regulations. This study was registered in the Chinese Clinical Trial Registry (ChiCTR) as ChiCTR1800017978 and in the Drug Trial Registration and Information Publication Platform (chinaDrugtrials.org.cn) as CTR20181466.

This study was performed at a Phase I Clinical Trial Center, Beijing Shijitan Hospital affiliated with Capital Medical University, Beijing, China, and approved by the Independent Ethics Committee of the participating institutions. All study participants provided signed informed consent and had the right to withdraw their consent at any time, without giving reason and without detriment.

### Study Design and Drug Administration

A randomized, open, single-dose, double-cycle, two-sequence, crossover PK-BE study was designed to be conducted in healthy Chinese subjects under fasting and postprandial conditions. The two-cycle study was separated by a 3-days washout period. A total of 108 healthy Chinese volunteers, both male and female, were enrolled in this study, and they were separated into two groups: 36 subjects in the fasting group and 72 subjects in the postprandial group. In each group, subjects were randomized into 2 treatment sequence groups: T-R or R-T (where T refers to the test tablet, and R refers to the reference tablet). The order in which subjects received the test and reference products during each cycle of the study was determined according to a randomization schedule generated by a biostatistician using SAS 9.4 software.

In the fasting group, each subject randomly received a single dose of the T or R tablet of 1 mg rasagiline after fasting overnight for 10 h. In the postprandial group, each subject received a single dose of the T or R tablet of 1 mg rasagiline with a high-fat and high-calorie breakfast (total energy 1000 kilocalories, 60% fat, 15% protein, 25% carbohydrate). The study drug was administered with 240 ml of water. Additional water intake was forbidden 1 hour before and after administration.

### Study Population

The estimation of the sample size was based on previous studies. The results that were obtained from a pilot study previously performed were considered. In the pilot study, the intraindividual variation (IIV) of the maximum observed serum concentration (C_max_) and the AUC were calculated to be approximately 37% and 15%, respectively, under postprandial conditions and 9% and 13%, respectively, under fasting conditions. In the studies of Van Rijswick YGJ, the IIV for the C_max_ of the reference product was 34.71% under fasting conditions (Van Rijswick et al.,2016). Considering also a 5% probability for type I errors and a power of at least 80%, the calculated sample size was 57. To this value, considering a 20% drop-out rate, 15 subjects were added; thus, 72 subjects were randomly entered into the postprandial group. Similarly, assuming that the maximum IIV of the main pharmacokinetic parameters is 25%, the calculated sample size was 36.

Healthy Chinese subjects aged 18 and over with a body mass index (BMI) between 19.0 kg/m^2^ and 28.0 kg/m^2^ were eligible for recruitment. Subjects needed to be in good health as determined by medical history, 12-lead electrocardiography (ECG), vital signs, physical examination, laboratory tests (haematology, blood biochemistry, blood pregnancy test, urinalysis, virological screening, alcohol breath test and other drugs of abuse) and nonsmoking status. Subjects were not allowed to take any medications or supplements throughout the study. To avoid hypertensive crisis, a diet rich in tyramine, such as wine and cheese, was prohibited. Eligible subjects did not have concomitant conditions or treatments that could have interfered with the study or the interpretation of its results. Women of childbearing age were eligible if they had a negative serum pregnancy test and were willing to use consistent and acceptable contraception for the duration of the study. Male subjects also agreed to use acceptable contraception during the study and for 6 months after the study. Enrolled subjects were not to have participated in another clinical trial within the last 3 months.

### Study Drugs

The reference tablet was rasagiline mesylate (1 mg Azilect^®^, Lot No. R81689, expiration date September 2019), produced by Teva Pharmaceutical Industries Ltd. The test tablet was rasagiline mesylate (1 mg, Lot No. 20170214, expiration date February 2020), manufactured by Changzhou Siyao Pharmaceuticals Co., Ltd., Jiangsu, China.

### Blood Sampling and Medical Supervision

Blood samples (4 ml each) for pharmacokinetic analysis of rasagiline were collected 1 hour pre-dose and 5, 10, 20, 30, and 45 minutes and 1.0, 1.33, 1.67, 2.0, 2.5, 3.0, 4.0, 6.0, 8.0, and 10.0 hours post-dose in the postprandial group. In the fasting group, blood samples were collected at the following time points: 1 hour pre-dose and 5, 10, 15, 20, 30 and 45 minutes and 1.0, 1.25, 2.0, 3.0, 4.0, 6.0, 8.0, 10.0 hours post-dose.

Subjects were confined to the study centre for continuous medical supervision until all PK blood samples were collected the next day after the second period of administration. Safety assessments included physical examination, vital signs, ECG recording, adverse event (AE) reporting, clinical laboratory measures and concomitant medication reporting. The number of subjects with AEs was summarized, coded, and classified according to the Medical Dictionary for Regulatory Activities (MedDRA) (version 20.0). AEs were graded according to the Common Terminology Criteria for Adverse Events (CTCAE) (version 4.03). Descriptive statistical analyses were applied.

### Chromatographic Conditions

Mobile A was 0.1% formic acid and 5 mmol/L ammonium acetate in water, and mobile B was 0.1% formic acid in acetonitrile. The flow rate was set as 0.5 ml/min, and the gradient was programmed as follows: 10% mobile B for 0.5 min; 10%–95% mobile B for 0.7 min; 95% mobile B maintained for 0.8 min; and 95%–10% mobile B for 0.1 min. The separation system was equilibrated with 10% mobile B for 1.0 min before the next analysis. An ultimate ACE 5 C18 (100 mm × 2.1 mm, 300A) analytical column was used to achieve favorable chromatographic separation. Both the temperature of the column and the autosampler were fixed at room temperature, and the injection volume was 5.0 μl.

### Mass Spectrometric Conditions

Quantitation of rasagiline and rasagiline-13C3 was achieved by multiple reaction monitoring (MRM) in positive ion mode in an electrospray ion source with the optimized parameters as follows: ion spray voltage of 5500 V, GS1 of 55 psi; GS2 of 55 psi; curtain gas of 30 psi; collision gas of 10 psi and source temperature of 550°C. Quantitation was performed by monitoring the transitions at m/z 172.0–117.0 for rasagiline and m/z 175.0–117.0 for rasagiline-13C3 with de-cluster voltage of 106 v and 116v as well as collision energy of 19 eV and 47 eV, respectively.

### Sample Pretreatment

First, 150 μl of human plasma was spiked with 50.0 μl of IS working solution containing 50.0 ng/ml of rasagiline-13C3. Then, 100 μL of sodium hydroxide solution with concentration of 0.1 mol/L was added to the mixture. After vortex for few seconds, 600 μl of methyl tert butyl ether was added, and followed by a at least 1 min shaking for extraction. Afterward, the solution was centrifuged at 3800 g for 10 min at 2–8°C. Finally, 450 μl of the supernatant was transferred into a new 96-well plate and evaporated to dryness under a nitrogen environment. The residue was reconstituted with 100 μl of solvent containing 0.1% formic acid in water for analysis.

### Assays of Rasagiline

Blood samples were centrifuged at 2500 g at 2–8°C for 10 minutes to obtain plasma samples. The plasma samples were stored at −80°C, which met the conditions for stability of the stored samples in the validation of methodology. Plasma concentrations of rasagiline were analysed using a validated liquid chromatography-mass spectrometry (LC-MS/MS) method. The linear range was 20.0–20000 pg/ml for rasagiline. The lower limit of quantification was 20.0 pg/ml. The precision range of each concentration quality control (QC) sample (% CV) was 3.5%–6.5%. The mean bias (%) from the theoretical concentration of QC samples was between -0.5% and 3.3% for rasagiline.

### Pharmacokinetic and Bioequivalence Analysis

PK parameters such as AUC_0–t_, AUC_0–∞_, C_max_, elimination half-life (T_1/2_), and time to achieve C_max_ (T_max_) were determined. The pharmacokinetic parameters of rasagiline were calculated with WinNonlin Version 6.4 by a noncompartment model. The AUC was calculated by the linear trapezoidal linear interpolation method. The experimental results were mainly analysed by descriptive statistics using SAS (SAS Institute, version 9.4). The main pharmacokinetic parameters after logarithmic transformation were analysed by a mixed-effect model. The level of significance was set at P < 0.05. The 90% confidence intervals (CIs) for the geometric mean ratios (GMRs) of C_max_, AUC_0–t_ and AUC_0–∞_ between the two formulations were obtained and then converted to the ratio scale by antilog transformation. If the 90% CI of the GMRs were completely within the range of 80.00–125.00%, bioequivalence was established.

## Results

### Subjects’ Demographic Characteristics

**Figure 1:**
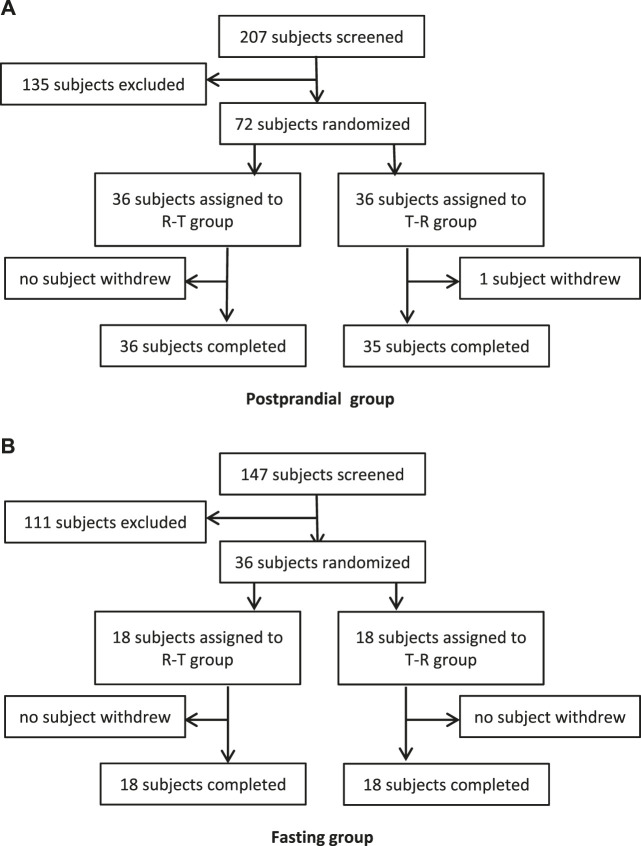
Study subjects disposition flow diagram.

In the fasting group, a total of 147 subjects were screened and assessed for eligibility, and 36 of them were enrolled. In the postprandial group, a total of 207 subjects were screened, and 72 subjects were enrolled. During the whole study, only 1 participant withdrew due to an AE of tachycardia after one administration. The flowchart of the subject distribution is shown in Figure 1. The demographic characteristics for each group are shown in Table 1.

**TABLE 1:** The demographic characteristics for each group.

Parameters	Fasting	Postprandial
N	36	72
Gender *n* (%)		
Male Female	20 (55.6%) 16 (44.4%)	62 (86.1%) 10 (13.9%)
Age (y)		
Mean ± SD Min–max	30.4 ± 7.55 18–45	30.7 ± 6.64 18–45
Weight (kg)		
Mean ± SD Min–max	66.12 ± 8.854 46.6–88.4	65.44±9.216 47.6–93.1
BMI (kg/m^2^)		
Mean ± SD Min–max	24.33 ± 2.223 19.8–27.3	23.15 ± 2.269 19.1–27.8

BMI, body mass index; Max, maximum; Min, minimum.

### Pharmacokinetic Calculations

**Figure 2:**
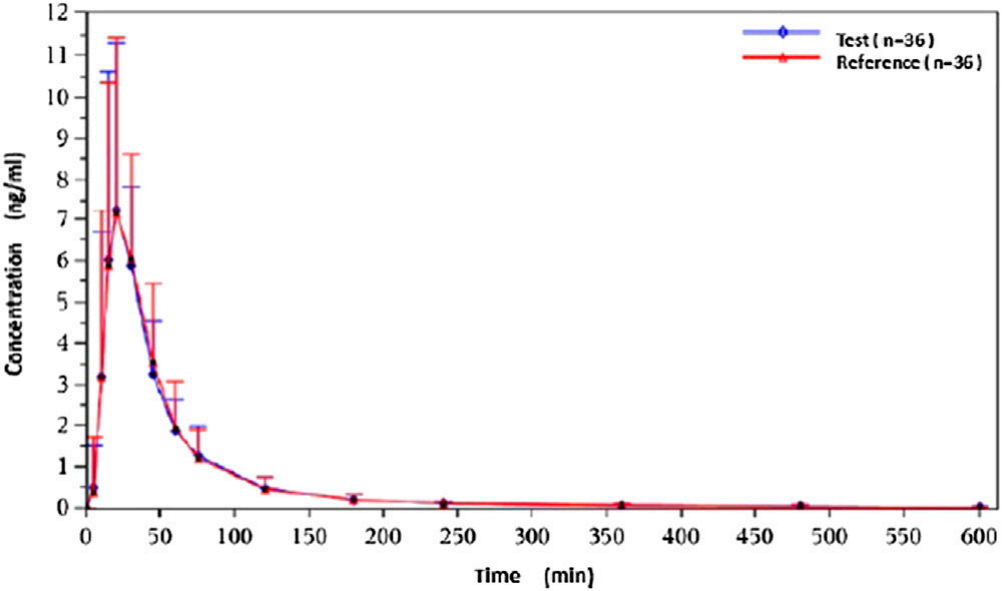
Concentration-time curves under fasting conditions.

**Figure 3:**
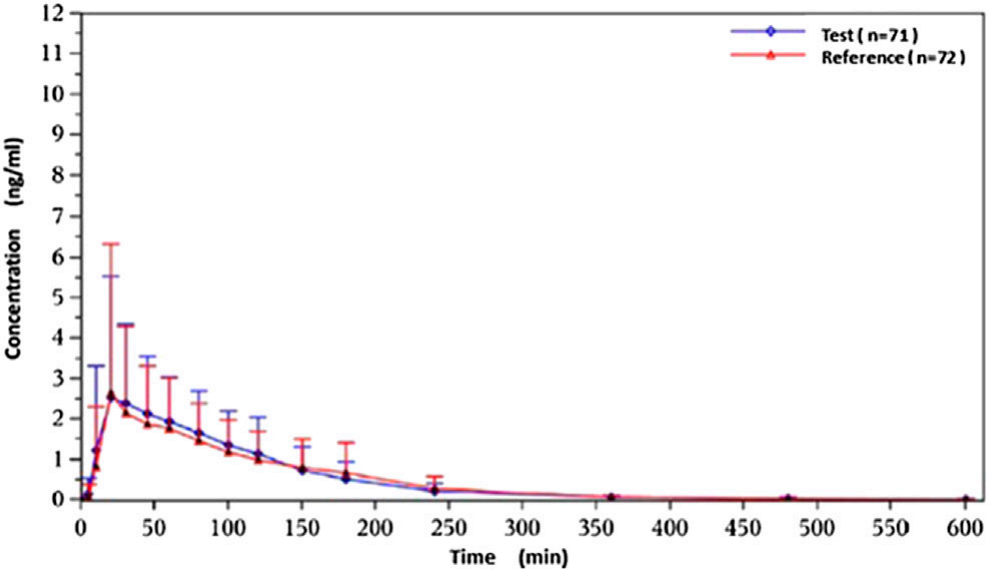
Concentration-time curves under postprandial conditions.

Under fasted and postprandial states, rasagiline PK parameters, measured using the geometric mean ratios (GMRs) of the AUC_0–t_, AUC_0–∞_, and C_max_, were similar between the test drug and the reference drug. The detailed PK parameters of the two rasagiline tablets using a single dose of 1 mg under both conditions are presented in Table 2 and Table 3. Rasagiline was rapidly absorbed by the gastrointestinal tract, with the median T_max_ occurring at 0.33 h post-dose under fasting conditions, while after a high-fat meal, the T_max_ of rasagiline was delayed to 1 h. The mean rasagiline plasma concentration-time profiles are depicted in Figure 2 and Figure 3.

**TABLE 2:** Pharmacokinetic parameters under fasting conditions.

Parameters	Mean ± SD (CV%)	
	Test (*N* = 36)	Reference (*N* = 36)
T_max_ (h)	0.33 (0.17, 0.75)	0.33 (0.17, 0.75)
C_max_ (ng/ml)	8.53 ± 3.60 (42.16)	8.87 ± 4.13 (46.52)
AUC_0–t_ (h ng/ml)	5.84 ± 1.66 (28.44)	5.91 ± 1.80 (30.40)
AUC_0–∞_ (h ng/ml)	5.99 ± 1.72 (28.65)	6.10 ± 1.90 (31.04)
t_½_ (h)	3.03 ± 1.51 (50.06)	3.49 ± 2.45 (70.20)

AUC_0–t_ indicates area under the concentration-time curve from 0 to t; AUC_0–∞_, area under the concentration-time curve from 0 to infinity; C_max_, maximum concentration; CV, coefficient of variation; t_½_, half-life of elimination; T max, time to C_max._ T_max_ was represented as median (min, max) (CV%).

**TABLE 3:** Pharmacokinetic parameters under postprandial conditions.

Parameters	Mean ± SD (CV%)	
	Test (*N* = 71)	Reference (*N* = 72)
T_max_ (h)	0.75 (0.17, 3.00)	1.00 (0.17, 3.00)
C_max_ (ng/ml)	4.33 ± 2.43 (56.18)	4.36 ± 2.98 (68.36)
AUC_0–t_ (h ng/ml)	4.93 ± 1.36 (27.60)	4.81 ± 1.38 (28.75)
AUC_0–∞_ (h ng/ml)	5.01 ± 1.39 (27.65)	4.89 ± 1.40 (28.67)
t_½_ (h)	1.82 ± 0.99 (54.37)	1.97 ± 3.02 (153.34)

AUC_0–t_ indicates area under the concentration-time curve from 0 to t; AUC_0–∞_, area under the concentration-time curve from 0 to infinity; C_max_, maximum concentration; CV, coefficient of variation; t_½_, half-life of elimination; T_max_, time to C_max._ T_max_ was represented as median (min, max) (CV%).

### Bioequivalence

Except for one subject who withdrew after the first cycle, a total of 71 subjects entered the BE assessment. The GMRs of the C_max_, AUC_0–t_ and AUC_0–∞_ were 97.84%, 99.60% and 99.12%, respectively, under fasting conditions, and the GMRs of the C_max_, AUC_0–t_ and AUC_0–∞_ were 102.89%, 103.41% and 103.25%, respectively, under postprandial conditions. All 90% confidence intervals (CIs) were within the range of BE from FDA guidelines (80%–125%). The results are shown in Table 4 and Table 5. A sensitivity analysis was performed to evaluate the influence of the data from the subject who dropped out and completed only the first cycle, and the results are presented in Table 6. The GMRs of the C_max_, AUC_0–t_ and AUC_0–∞_ were 102.86%, 103.39% and 103.22%, respectively. These 90% CIs from the sensitivity analysis were within the same range of BE.

**TABLE 4:** Bioequivalence under fasting conditions.

PK parameter	GM and GMR			IIV%	90%CI
	T (*N* = 36)	R (*N* = 36)	GMR (%)		
C_max_ (ng/ml)	7.820	7.992	97.84	26.30	88.26–108.46
AUC_0–t_ (h ng/ml)	5.597	5.620	99.60	14.15	94.16–105.35
AUC_0–∞_ (h ng/ml)	5.740	5.791	99.12	14.58	93.55–105.01

GMR refers to the geometric mean ratio of the test over reference pharmacokinetic metric. IIV refers to the intraindividual variation.

**TABLE 5:** Bioequivalence under postprandial conditions.

PK parameter	GM and GMR			IIV%	90%CI
	T (*N* = 71)	R (*N* = 71)	GMR (%)		
C_max_ (ng/ml)	3.759	3.656	102.89	52.89	89.54–118.23
AUC_0–t_ (h ng/ml)	4.762	4.614	103.41	12.45	99.88–107.07
AUC_0–∞_ (h ng/ml)	4.838	4.696	103.25	12.94	99.59–107.05

GMR refers to the geometric mean ratio of the test over reference pharmacokinetic metric. IIV refers to the intraindividual variation.

**TABLE 6:** Sensitivity analysis under postprandial conditions.

PK parameter	GM and GMR			IIV%	90%CI
	T (*N* = 71)	R (*N* = 72)	GMR (%)		
C_max_ (ng/ml)	3.759	3.656	102.86	52.89	89.59–118.11
AUC_0–t_ (h ng/ml)	4.762	4.614	103.39	12.45	99.86–107.03
AUC_0–∞_ (h ng/ml)	4.838	4.696	103.22	12.94	99.57–107.01

GMR refers to the geometric mean ratio of the test over reference pharmacokinetic metric. IIV refers to the intraindividual variation.

The main pharmacokinetic parameters (C_max_ and AUC) related to drug exposure were logarithmically transformed and tested by multivariate analysis of variance (ANOVA). The results of the ANOVA test are presented in Table 7. There were no significant differences in the main parameters among different sequences and preparations. Except for LNC_max_ under postprandial conditions, the main parameters were significantly different in different periods under both conditions. It does not affect the establishment of bioequivalence.

**TABLE 7:** Results of ANOVA test of 2 formulations.

Parameters	P (fasting)			P (postprandial)		
	LnC_max_	LnAUC_0–t_	LnAUC_0–∞_	LnC_max_	LnAUC_0–t_	LnAUC_0–∞_
Sequence	0.8533	0.9330	0.9452	0.6270	0.2601	0.2885
Formulation	0.7227	0.9041	0.7969	0.7339	0.1117	0.1436
Period	<0.0001	<0.0001	<0.0001	0.3362	<0.0001	<0.0001

ANOVA, analysis of variance; p < 0.05 was considered to be statistically significant.

### Safety and Tolerability

Treatment-emergent AEs are presented in Table 8. Data from 108 subjects were included in this safety evaluation. Of the 36 subjects enrolled in the fasting group, all completed the two-cycle study. Of these, 10 subjects (27.8% of 36) reported 17 drug-related AEs. Of the 17 AEs, 6 subjects (16.7% of 36) experienced 6 post-dose AEs related to the test product, and 6 subjects (16.7% of 36) experienced 11 post-dose AEs related to the reference product. In terms of the severity of AEs, all AEs were classified as level 1 to level 2 (NCI CTCAE 4.03), and there were no AEs of level 3 or above. No serious AEs (SAEs) occurred during the treatment.

**TABLE 8:** Summary of treatment-emergent adverse events.

Parameter	Fasting conditions			Postprandial conditions		
	T	R	Total	T	R	Total
No. of subjects dosed	36	36	—	71	72	—
No. (%) of subjects with at least 1 TEAE	6 (16.7)	6 (16.7)	10 (27.8)	10 (14.1)	10 (13.9)	19 (26.4)
No. of TEAEs	6	11	17	14	23	37
Grade 1	5	7	12	11	17	28
Grade 2	1	4	5	2	5	7
≥Grade 3	0	0	0	1	1	2
No. of subjects discontinued due to TEAE	0	0	0	1	0	1
Adverse event [no. (%) of subjects, no. of AEs]	—	—	—	—	—	—
Hypertriglyceridemia	3 (8.3) 3	1 (2.8) 1	—	3 (4.2) 3	4 (5.6) 4	—
Hyperkalemia	0 (0) 0	0 (0) 0	—	1 (1.4) 1	0 (0) 0	—
Monocytopenia	0 (0) 0	0 (0) 0	—	2 (2.8) 2	0 (0) 0	—
Eosinophil count increased	0 (0) 0	0 (0) 0	—	0 (0) 0	4 (5.6) 4	—
Proteinuria	0 (0) 0	0 (0) 0	—	2 (2.8) 2	0 (0) 0	—
Positive acetone in the urine	0 (0) 0	0 (0) 0	—	0 (0) 0	1 (1.4) 1	—
Urine leukocytes increased	1 (2.8) 1	1 (2.8) 1	—	0 (0) 0	1 (1.4) 1	—
Alanine aminotransferase increased	0 (0) 0	1 (2.8) 1	—	0 (0) 0	0 (0) 0	—
Blood basophils increased	0 (0) 0	1 (2.8) 1	—	0 (0) 0	0 (0) 0	—
Aspartate aminotransferase increased	0 (0) 0	1 (2.8) 1	—	0 (0) 0	0 (0) 0	—
Blood conjugated bilirubin increased	0 (0) 0	2 (5.6) 2	—	0 (0) 0	2 (2.8) 2	—
Blood unconjugated bilirubin increased	0 (0) 0	1 (2.8) 1	—	1 (1.4) 1	2 (2.8) 2	—
Blood total bilirubin increased	0 (0) 0	1 (2.8) 1	—	1 (1.4) 1	2 (2.8) 2	—
Diarrhea	1 (2.8) 1	1 (2.8) 1	—	0 (0) 0	0 (0) 0	—
Hypertension	1 (2.8) 1	1 (2.8) 1	—	2 (2.8) 2	2 (2.8) 2	—
Rapid pulse	0 (0) 0	0 (0) 0	—	1 (1.4) 2	2 (2.8) 3	—
Upper respiratory tract infection	0 (0) 0	0 (0) 0	—	0 (0) 0	1 (1.4) 1	—
Eczema	0 (0) 0	0 (0) 0	—	0 (0) 0	1 (1.4) 1	—

In the postprandial group, 72 subjects were recruited, and 71 subjects completed both periods of the study. A total of 19 subjects (26.4% of 72) reported 37 drug-related AEs. Ten subjects (14.1% of 71) experienced 14 post-dose AEs related to the test product, and 10 subjects (13.9% of 72) experienced 23 post-dose AEs related to the reference product. Among these AEs, there were two events of hypertension, severity level 3, related to T and R tablet administration. In this group, one subject withdrew due to tachycardia. No SAEs or deaths were observed during the study.

During the last scheduled visit, all AEs were resolved in both groups.

## Discussion

At present, there is no uniform regulation on whether males and females should be included in BE study. In many countries, BE studies do not even include female subjects. Because the guidelines of BE study do not make specific requirements on gender ratio, so in this trial, the gender ratio was not balanced.

In addition to a BE study under fasting conditions, the US FDA and National Medical Products Administration (NMPA) currently recommend that a BE study under fed conditions should be conducted for all orally administered drug products submitted as an abbreviated new drug application (ANDA), with only a few specific exceptions: when the drug substance is considered a BCS class I drug and dissolution, solubility, and permeability data support a biowaiver of *in vivo* BE testing, when the DOSAGE AND ADMINISTRATION section of the reference listed drug (RLD) label states that the product should be taken only on an empty stomach, or when the RLD label does not make any statements about the effect of food on absorption or administration (FDA, 2002; National Medical Products Administration, 2016). Rasagiline is a highly soluble, low-permeability drug and is therefore classified as a BCS class III product. According to the RLD label, rasagiline can be administered with or without food because the AUC is not significantly affected by food (Teva Pharmaceuticals USA, 2018). Therefore, this BE study for rasagiline was conducted under both fasting and postprandial conditions. The main PK parameters (C_max_ and AUC) of the test drug and the reference drug had similar performances under both fasting and postprandial conditions. The 90% CIs of the GMRs for the AUC and C_max_ of the two tablets were within the BE range.

As shown above, rasagiline was rapidly absorbed by the gastrointestinal tract, with the T_max_ occurring between 0.17 and 0.75 hours after a single 1 mg dose under fasting conditions. After a high-fat meal, the T_max_ of rasagiline was delayed from 0.33 hours under fasting conditions to 1 hour under postprandial conditions. This result is somewhat different from that of previous studies which demonstrated that food did not affect the T_max_ of rasagiline, although the C_max_ and exposure (AUC) were decreased by approximately 60% and 20%, respectively, when the drug was taken with a high-fat meal (Chen and Swope, 2005; Teva Pharmaceuticals USA, 2018). In our study, we also found that while a high-fat meal had significant effects on the absorption rate of rasagiline, it had minor effects on the absorption amount. Compared with fasting conditions, the C_max_ of rasagiline measured after a high-fat meal decreased significantly (4.36 ± 2.98 vs. 8.87 ± 4.13), and the AUC decreased slightly (4.89 ± 1.40 vs. 6.10 ± 1.90), which was consistent with prior literature.

A study showed that, in most cases, increased bioavailability with food resulted in a decrease in the IIV of the AUC. While food had a negative effect on a drug's bioavailability, the administration of the drug with food resulted in greater IIV than administration without food (Kang and Ratain, 2010). In our study, we found that when the drug was administered with a high-fat meal, corresponding to the decrease in C_max_, there was a significant increase in the coefficient of variation (CV) (26.3% under fasting conditions vs 52.89% under postprandial conditions).

As stated above, administration with food can influence the absorption and systemic exposure of rasagiline. The effect of food on oral bioavailability results from a complex interplay of drug, formulation, food components, and gastric and intestinal physiology (e.g., gastrointestinal pH, gastric emptying, intestinal transit) (Fleisher et al.,1999; FDA, 2002; Welling, 1989). The difference in PK parameters obtained may have resulted from differences in subjects, sample sizes, sample detectors, or other unknown factors. Additional considerations such as interindividual variability may also contribute to this difference (Elgart et al.,2019).

The results from previous studies suggested that rasagiline was a highly variable drug (HVD), which was consistent with our study (Kang and Ratain, 2010; Van Rijswick et al., 2016). In 2016, a randomized, four-period, two-sequence, single-dose, replicate-crossover BE study of rasagiline was conducted in 30 healthy, adult male volunteers under fasting conditions by Van Rijswick YGJ. In the studies of Van Rijswick YGJ, the IIV for the C_max_ of the reference product was 34.71% (Van Rijswick et al.,2016). In our study, after coadministration with food, the IIV for the C_max_ of rasagiline was up to 52.89% under postprandial conditions, much higher than the data from the studies of Van Rijswick YGJ under fasting conditions. Traditionally, statistical analysis of BE study data is performed by obtaining the average bioequivalence (ABE) approach via a two-way crossover design. Two products are deemed bioequivalent when the 90% CIs of the GMRs for the AUC and C_max_ fall within the limits of 80%–125% by the ABE. Establishing BE for HVDs is challenging, as a high IIV requires a dramatically larger sample size. Since 2006, the FDA has accepted a reference-scaled average bioequivalence (RSABE) approach for HVDs. Using the RSABE approach, for a BE study, researchers can use either a partial replicate (three-way crossover, RTR, RRT, or TRR) or a full replicate (four-way crossover, RTRT or TRTR) design, and the BE limits are broadened to greater than 80%–125% (Davit et al.,2012; European Medicine Agency, 2010; FDA, 2014; FDA, 2011). This BE study has a two-way crossover design, meeting the ABE criteria that were also required by the NMPA for HVDs (National Medical Products Administration, 2018). Because rasagiline is an HVD, the BE between two preparations of rasagiline was established by expanding the sample size of the postprandial group to 72 individuals.

The ANOVA test suggested the variation between periods was significant. But it does not mean the two preparations are not equivalent. It may be influenced by season, room temperature, air pressure and humidity, visceral accumulation or damage, drug enzyme induction or resistance and so on. In this trail, a 3 days washout period is sufficient, much longer than the seven elimination half-lives of rasagiline.

In addition to having PK BE, both types of rasagiline were well tolerated, with no significant differences between safety profiles. And there was no significant difference in safety between both fasting group and postprandial group. The occurrence and severity of AEs in our study were similar to those from previous studies and described under the rasagiline label.

## Conclusion

Data from this study show that there is no difference in absorption rate and exposure between the two preparations of rasagiline. All subjects showed good tolerance of both preparations without any serious or unexpected adverse reactions. According to the relevant NMPA guidelines, the two tablets were considered bioequivalent for a single dose under fasting and postprandial conditions.

## Data Availability Statement

The raw data supporting the conclusions of this article will be made available by the authors, without undue reservation.

## Ethics Statement

The studies involving human participants were reviewed and approved by the Independent Ethics Committee of Bejing Shijitan Hospital. The patients/participants provided their written informed consent to participate in this study.

## Author Contributions

All authors critically reviewed the manuscript and contributed to conception and design of the protocol. YJL, XW, and HB had involved in drafting the manuscript or revising it critically for important intellectual content.

## Funding

This work was supported by the Ministry of Science and Technology of the People's Republic of China (No. 2017ZX09304026) and Youth fund of Beijing Shijitan Hospital Affiliated to Capital Medical University (No. 2018-q18).

## Conflict of Interest

YT, YS, JW, QQ, XF, and DZ were employed by the company Changzhou Siyao Pharmaceuticals Co., Ltd.The remaining authors declare that the research was conducted in the absence of any commercial or financial relationships that could be construed as a potential conflict of interest.The authors declare that this study received funding from Changzhou Siyao Pharmaceuticals Co., Ltd. The funder had the following involvement with the study: study design, decision to publish and preparation of the manuscript.
